# Revisiting Old Ionophore Lasalocid as a Novel Inhibitor of Multiple Toxins

**DOI:** 10.3390/toxins12010026

**Published:** 2020-01-01

**Authors:** Nassim Mahtal, Yu Wu, Jean-Christophe Cintrat, Julien Barbier, Emmanuel Lemichez, Daniel Gillet

**Affiliations:** 1 Service d’Ingénierie Moléculaire des Protéines (SIMOPRO), CEA, Université Paris-Saclay, F-91191 Gif-sur-Yvette, France; nassim.mahtal@inserm.fr (N.M.); yu.wu@cea.fr (Y.W.); 2 Service de Chimie Bio-organique et Marquage (SCBM), CEA, Université Paris-Saclay, F-91191 Gif-sur-Yvette, France; jean-christophe.cintrat@cea.fr; 3 Unité des Toxines Bactériennes, Institut Pasteur, ERL 6002, F-75015 Paris, France; emmanuel.lemichez@pasteur.fr

**Keywords:** lasalocid, toxin, Golgi

## Abstract

The ionophore lasalocid is widely used as a veterinary drug against coccidiosis. We found recently that lasalocid protects cells from two unrelated bacterial toxins, the cytotoxic necrotizing factor-1 (CNF1) from *Escherichia. coli* and diphtheria toxin. We evaluated lasalocid’s capacity to protect cells against other toxins of medical interest comprising toxin B from *Clostridium difficile*, Shiga-like toxin 1 from enterohemorrhagic *E. coli* and exotoxin A from *Pseudomonas aeruginosa*. We further characterized the impact of lasalocid on the endolysosomal and the retrograde pathways and organelle integrity, especially the Golgi apparatus. We found that lasalocid protects cells from all toxins tested and impairs the drop of vesicular pH along the trafficking pathways that are required for toxin sorting and translocation to the cytoplasm. Lasalocid also has an impact on the cellular distribution of GOLPH4 and GOLPH2 Golgi markers. Other intracellular trafficking compartments positive for EEA1 and Rab9A display a modified cellular pattern. In conclusion, lasalocid protects cells from multiple deadly bacterial toxins by corrupting vesicular trafficking and Golgi stack homeostasis.

## 1. Introduction

Many bacteria, viruses, parasites and bacterial and plant protein toxins enter cells by following a number of cell endocytic pathways. Some pathogens reside in a vacuole build of their own from different vesicular compartments. Others gain access to the cytoplasm by escaping from specific vesicular compartments along vesicular trafficking pathways. The endo-lysosomal route comprises early endosomes, which progressively maturate and merge with other vesicles into multi-vesicular bodies and give rise to late endosomes [1]. Maturation of endocytic compartments and protein sorting processes are largely driven by luminal acidification that is also exploited by pathogens and toxins to translocate into the cytosol [2]. This is the case for diphtheria toxin (DT) and *Clostridium difficile* glucosylating toxins comprising toxin B (TcdB). Other toxins traffic through the retrograde pathway, from the early endosomes to the Golgi apparatus and then the endoplasmic reticulum (ER). Exotoxin A (ETA) from *Pseudomonas aeruginosa* and Shiga-like toxins such as Stx1 from pathogenic *E. coli* belong to this category [3].

Ionophores are chemicals that disrupt ion gradients across biological membranes by facilitating ion diffusion through these membranes. Several antimicrobial drugs for veterinary use are ionophores [4]. One of the best documented example is probably monensin, a cationic ionophore with Na^+^ and K^+^ specificity used as an antibiotic in ruminant feed [5].

Lasalocid is another carboxylic ionophore with a broader ion specificity than monensin including the capacity to bind monovalent and divalent cations [5,6,7]. Produced by *Streptomyces lasaliensis*, it is widely used as a feed additive in cattle and poultry industries to control coccidiosis, a disease of the intestinal track caused by *Eimeria* protozoa [8,9]. Although lasalocid kills Gram-positive bacteria, it also affects other pathogens, and could help to fight mastitis-causing microbes belonging to *Streptococcus* and *Staphylococcus* subgroups, and *Trypanosoma brucei* infections [10,11]. Overall, lasalocid is mainly used in the industry to enhance productivity and prevent costly deadly coccidiosis [12].

Sandvig and colleagues have shown in 1982 that a brominated analog of lasalocid (BrX-537A) can protect cells from DT [13], and previous work in our laboratory showed that lasalocid hinders cell intoxication by DT and the cytotoxic necrotizing factor-1 (CNF1) from extraintestinal strains of pathogenic *E. coli* [14]. However, cell biology effects following cell exposure to this compound are poorly described.

The objective of this study was to evaluate the protective effect of lasalocid against toxins other than DT and CNF1 and to better characterize its effects on intracellular compartments. Here we report that lasalocid protects cells from Stx1, ETA and TcdB. By monitoring specific markers of various organelles, we show a disorganizing effect of lasalocid on the Golgi apparatus, the early endosomes and the lysosomes. This correlates with recorded broad alterations of the physicochemical properties of intracellular compartment [15,16]. Taken together, these findings unveil a broad anti-bacterial toxin effect of lasalocid.

## 2. Results

### 2.1. Lasalocid Effects on Cell Intoxication by TcdB, Stx1 or ETA

**Figure 1:**
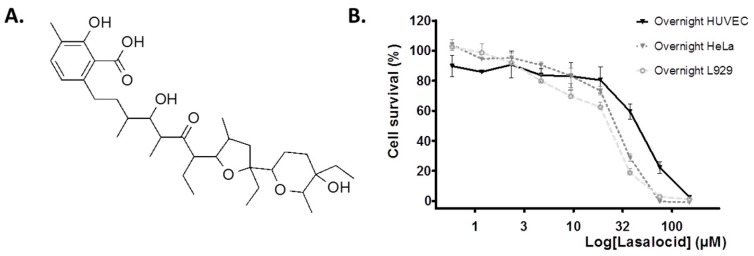
Lasalocid structure and cytotoxicity. (**A**) Chemical structure of lasalocid. (**B**) HeLa cells, L929 cells or human umbilical vein endothelial cells (HUVECs) were incubated with lasalocid at the indicated concentrations, DMSO 10% or left untreated (controls) overnight, before incubation with resazurin. Fluorescent signal, reflecting viability, was measured and data were normalised (100% viability corresponding to untreated cells and 0% to treated cells with 10% DMSO).

The chemical structure of carboxylic ionophore lasalocid is depicted in Figure 1A. To determine working concentrations in cell protection experiments against toxins, we first evaluated the intrinsic cytotoxicity of lasalocid on a cell line (HeLa) and primary human umbilical vein endothelial cells (HUVECs). We measured cell viability after overnight incubations of cells with lasalocid (Figure 1B). After normalization of the data, we observed a better tolerance of HUVECs, and more than 80% of viability for lasalocid concentrations ≤ 20 µM.

**Figure 2:**
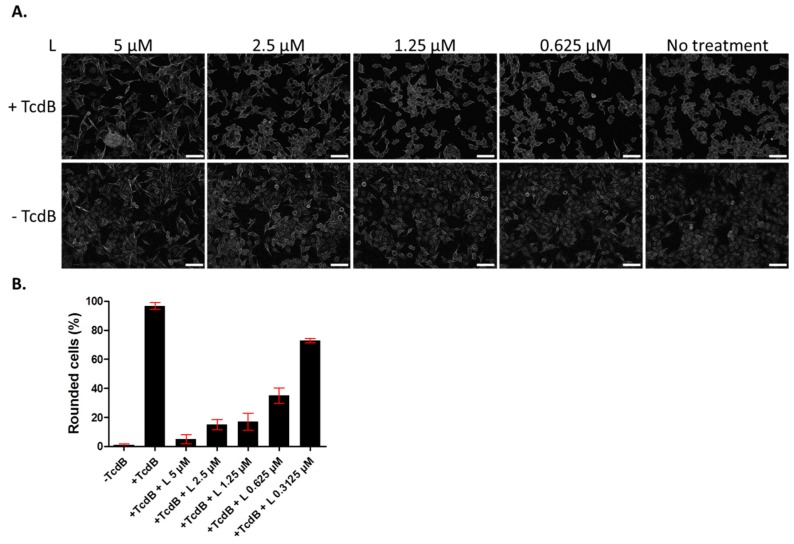
Lasalocid inhibits toxin B (TcdB) induced-HUVEC rounding. HUVECs were treated overnight with lasalocid (L) in the presence or not of TcdB, or left without TcdB or lasalocid (control conditions). The following day, cells were fixed and imaged with a Cytation 5 reader (objective 10×) (**A**). Rounded cells were then counted and normalized to the number of total cells (**B**). Scale bar: 200 µm.

Since we showed previously that lasalocid protects cells from CNF1 and DT cytotoxicity [14], we investigated whether it might give a broader antitoxin protection of cells by acting against TcdB. The latter is an unrelated toxin trafficking through the endo-lysosomal pathway, that is produced by the pathogen *C. difficile* responsible for nosocomial pseudomembranous colitis. TcdB acts by glucosylating the small GTPases Rac1, RhoA and Cdc42, which leads to actin cytoskeleton disruption and a rounding of cells. HUVECs were incubated overnight with TcdB, in the presence or absence of lasalocid, and cell phenotype was observed the next day by light microscopy (Figure 2). All cells treated with TcdB alone displayed a round phenotype. Addition of lasalocid prevented TcdB effects in a dose-dependent manner.

**Figure 3:**
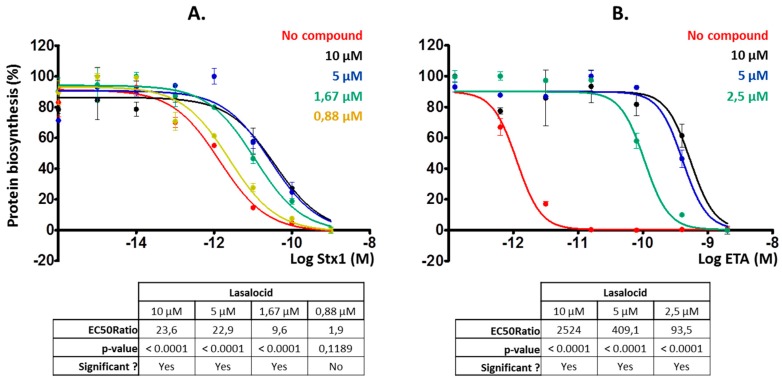
Lasalocid decreases Stx1 and exotoxin A (ETA) inhibitory effect on protein biosynthesis. Cells were preincubated for 3 h with lasalocid at indicated concentrations, or left untreated, and escalating doses of Stx1 (**A**) or ETA (**B**) were added and incubated overnight. The following day, medium was replaced by [^14^C]-Leu-containing medium and signal was read 6 h later to measure protein biosynthesis. HeLa cells were used for Stx1 experiments, and L929 cells for ETA for higher sensitivity to the toxin. EC_50_ ratios (comparing EC_50_ of each treated condition with the untreated control) calculated by Prism software are indicated.

Then, we investigated whether lasalocid could protect cells from ETA and Stx1 that traffic through the retrograde pathway, translocate into the cytoplasm from the ER and inhibit protein synthesis. We chose HeLa cells for Stx1 experiments and L929 cells for the ones involving ETA because these cells are more sensitive to ETA [17] and allowed us to reduce the quantity of toxin to use. Lasalocid strongly protected cells from Stx1 and ETA (Figure 3A,B), reducing toxicity more than 20-fold and 2500-fold, respectively. Hence, lasalocid has broad-spectrum antitoxin properties, acting on unrelated toxins trafficking either through the endo-lysosomal pathway or the Golgi-ER retrograde pathway.

### 2.2. Lasalocid Affects Lysosomal Acidification and Protein Degradation as Well as the Phenotype of the Golgi Apparatus

**Figure 4:**
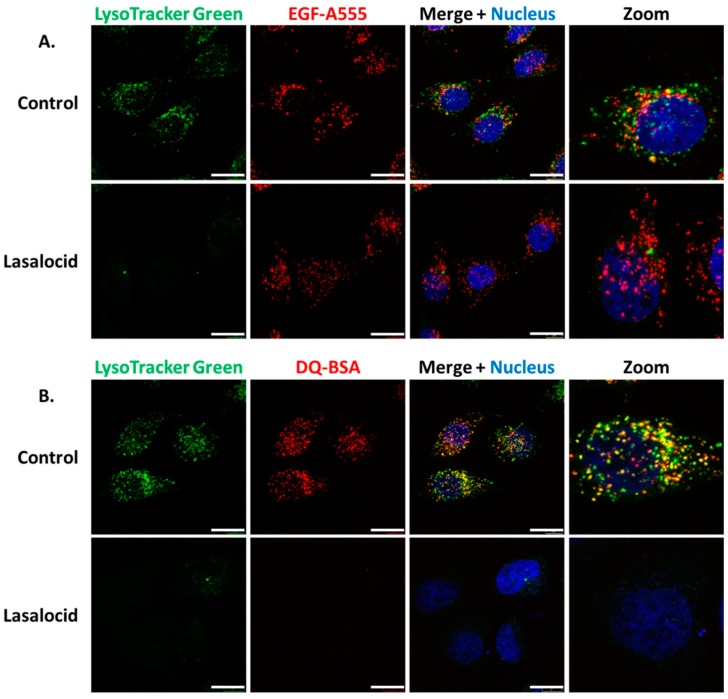
Lasalocid affects vesicular acidification but not endocytosis of epidermal growth factor (EGF) and blocks bovine serum albumin (BSA) degradation. (**A**) HeLa cells were treated with 10 µM of lasalocid, or left untreated (control), over 6 h. LysoTracker Green (acidic compartment staining), EGF-A555 and H33342 (nuclear staining) were added after 30 min. Cells were washed and images were taken with a confocal microscope. (**B**) HeLa cells were processed according to the previous protocol with DQ Red bovine serum albumin (DQ-BSA) instead of EGF-A555. Scale bar: 20 µm.

Following receptor-mediated endocytosis CNF1, DT and TcdB exploit the endolysosomal pathway to reach the cytoplasm [18,19,20,21]. Thus, we investigated the effect of lasalocid on this trafficking pathway. HeLa cells were treated with lasalocid for 6 h. During the last 30 min, different fluorescent probes were added to the cells. This comprises LysoTracker Green, which accumulates in lysosomes due to their low pH, and Alexa555-labelled epidermal growth factor (EGF-A555), which enters cells through clathrin-dependent endocytosis (Figure 4A). The EGF/EGF receptor (EGFR) complex traffics through the endo-lysosomal pathway, and EGFR can be either recycled back to the plasma membrane or reach lysosomes together with EGF where acidic-based protein degradation occurs. After lasalocid treatment, no LysoTracker signal could be detected. In contrast, the EGF-A555 signal remained identical in treated and untreated control cells. These results suggest that lasalocid-treated cells can still internalize proteins (here shown with EGF) but have an alteration of lysosomes or/and lysosomal acidification as reflected by the loss of LysoTracker staining. Furthermore, we exposed cells to lasalocid for 6 h and added DQ Red bovine serum albumin (DQ-BSA). DQ-BSA is labelled with a fluorophore that is quenched when the protein is intact and emits fluorescence after degradation at low pH (i.e., in lysosomes) (Figure 4B). No DQ-BSA signal could be recorded in lasalocid-treated cells, indicating a defect of low pH-dependent degradation of BSA.

Together, these results suggest that lasalocid does not block endocytosis but alters acidification of the endolysosomal pathway required for protein degradation.

**Figure 5:**
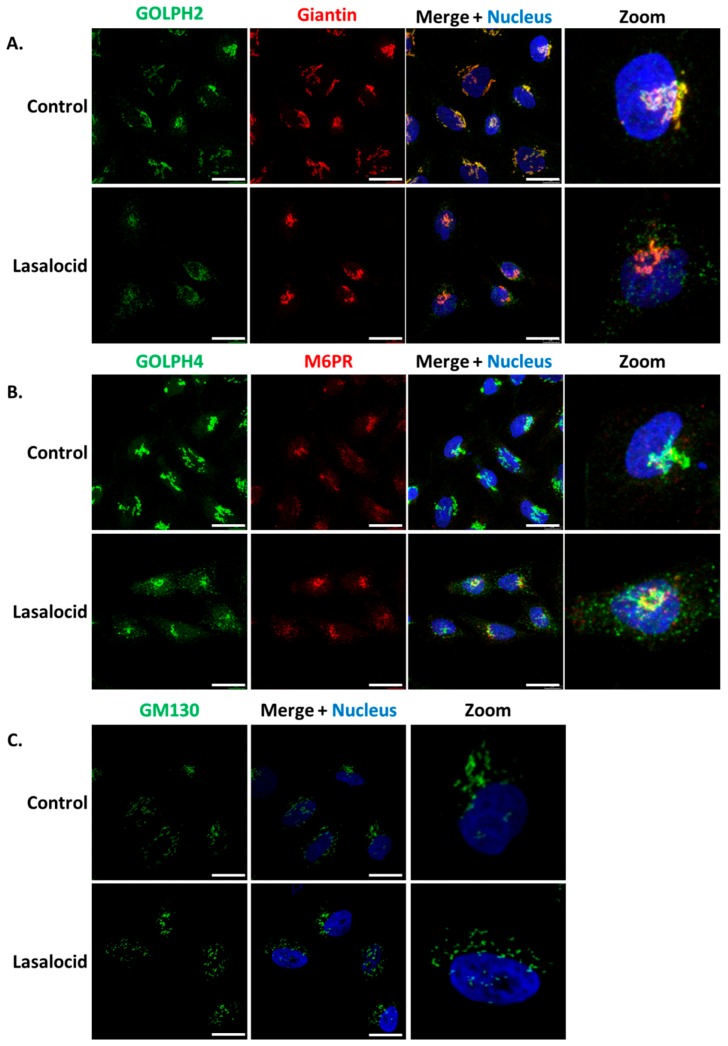
Lasalocid induces morphological changes of the Golgi apparatus. HeLa cells were treated 6 h with 10 µM lasalocid or left untreated (control), fixed and labelled with antibodies directed against (**A**) GOLPH2 and Giantin, (**B**) GOLPH4 and M6PR, (**C**) GM130. In all cases, H33342 was used to label the nucleus. Scale bar: 20 µm.

ETA and Stx1 travel from early endosomes to the Golgi apparatus, and then reach the endoplasmic reticulum in order to get access to the cytoplasm. Based on the capacity of lasalocid to protect cells against these toxins, we investigated the impact of this compound on the distribution of several Golgi makers, namely GOLPH2, GOLPH4, GM130, giantin and the trans-Golgi Mannose-6-Phosphate Receptor (M6PR). In control cells, the markers of Golgi sub-compartments form condensed and perinuclear structures, as expected (Figure 5). However, GOLPH2 and GOLPH4 displayed a scattered cytoplasmic localization upon lasalocid treatment. Interestingly, giantin, GM130 and M6PR localization patterns remain unaffected. Therefore, lasalocid partially affects Golgi protein localization.

### 2.3. Lasalocid Affects Early and Recycling Endosomes

**Figure 6:**
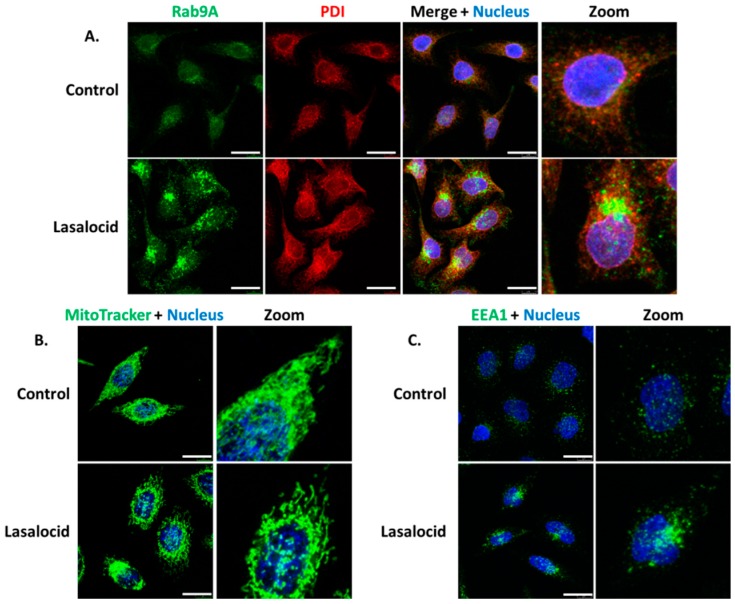
Vesicles and organelles labelling. The same experimental conditions than in Figure 5 were used for (**A**–**C**). Lasalocid-treated and untreated cells were labelled with anti-Rab9A antibodies (**A**) and anti-EEA1 antibodies (**C**). In (**B**), lasalocid-treated and untreated HeLa cells were incubated with MitoTracker for 30 min, washed and then imaged using a confocal microscope. Scale bar: 20 µm.

Since lasalocid affects the pH of endolysosomal vesicular compartments and the phenotype of the Golgi apparatus, we investigated its effect on other organelles by monitoring proteins implicated in cellular trafficking. Endoplasmic reticulum (PDI labelling) and mitochondria (MitoTracker labelling) are also affected by lasalocid (Figure 6A,B). Lasalocid modifies recycling vesicles and early endosomes as indicated by immunostaining of their respective markers, Rab9A and EEA1 (Figure 6A,C). Both types of compartments appear more condensed with changes in their distribution patterns.

### 2.4. Lasalocid Affects Autophagy

**Figure 7:**
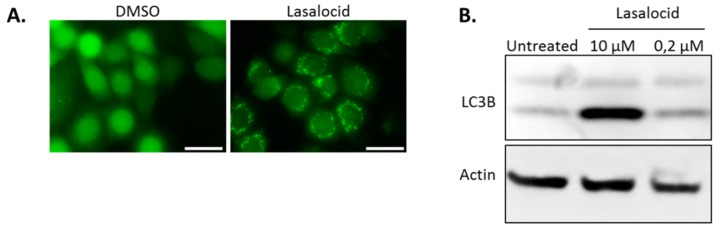
Lasalocid modulates autophagy by increasing LC3B. (**A**) HeLa cells stably expressing GFP-LC3B were treated with 2.5 µM lasalocid or the corresponding dilution of DMSO (lasalocid vehicle) for 5 h, and then imaged by fluorescence microscopy. (**B**) HeLa cells were treated 6 h with 10 µM or 0.2 µM lasalocid, or DMSO alone, and lysed to perform an SDS-PAGE and Western blot with antibody detection of LC3B and actin, actin serving as cell lysate loading control. Scale bar: 20 µm.

Autophagy is a cellular process leading to vesicle fusion with lysosomal compartments and degradation of vesicular content. Because lasalocid affects vesicles’ pH and trafficking, we investigated its impact on autophagy. HeLa cells expressing the fluorescent autophagy marker GFP-LC3B were treated 6 h with lasalocid (or DMSO as a control), and imaged by fluorescence microscopy (Figure 7A). In control cells, GFP-LC3B fluorescence appeared diffused throughout cells with a stronger labelling in the nuclei than in the cytoplasm. In contrast, in the lasalocid-treated cells, GFP-LC3B gave a pattern of intense perinuclear dots. In addition, SDS-PAGE and Western blots were performed on HeLa cells with antibodies targeting the LC3B isoform of LC3 and actin as a loading control (Figure 7B). The results show that lasalocid induces the accumulation of the faster migrating form of LC3B called LC3B-II. LC3B-II, a lipidated form of LC3B, is a marker of autophagophores. This effect is dose dependent. In conclusion, lasalocid affects autophagy, either by increasing autophagic vesicles formation or by lowering their degradation.

## 3. Discussion

Altogether, our results show that lasalocid, a widely used veterinary drug, protects cells from Stx1, ETA and TcdB, in addition to DT and CNF1 [14]. This antitoxin effect occurs at low micromolar concentrations that do not exhibit any cytotoxicity. Lasalocid impairs the endolysosomal route, regulates autophagy, and affects partially the Golgi apparatus since GOLPH2 and GOLPH4 are redistributed, but not GM130, M6PR and giantin. Alteration of vesicular acidification by lasalocid also has an impact on early and recycling endosomes, which could explain its broad anti-toxin properties. Hence, lasalocid is a useful tool to study protein trafficking, organelles alteration and toxin effects, due to its broad antitoxin activity and low toxicity to human cells.

Several intracellular compartments are affected by lasalocid: early endosomes, lysosomes, recycling and autophagic vesicles, mitochondria and the Golgi apparatus. These compartments are implicated in various intracellular trafficking pathways: the endolysosomal and recycling routes, the retrograde pathway and autophagy. These effects are accompanied by a blockage of endocytosed protein degradation in lysosomes (shown by BSA degradation assay), consistent with results from Grinde in 1983 in rat hepatocytes, and Reijngoud et al. in 1976 in rat liver lysosomes [16,22]. Besides, clathrin-dependent endocytosis is preserved as shown by EGF internalization assay. Furthermore, the autophagy process that involves the endosome to lysosome pathway is also altered as shown by increased LC3B-II and modification of Rab9A distribution pattern. LC3B accumulates in autophagophores [23], and Rab9A is involved in endosomal sorting, lysosomes’ biogenesis, retrograde transport and autophagy regulation [24,25,26,27,28]. A similar impact on LC3B-II has been described recently by Kim et al. in 2017 [29]: lasalocid seems to prevent autophagocyted material to be degraded, but not the formation of autophagosomes. 

Although such a broad effect on organelles could have suggested poor tolerance of lasalocid by cells, we did not observe significant cytotoxicity after 24 h exposure at the concentration causing these modifications. This questions whether lasalocid could have a realistic antitoxin therapeutic potential. Lasalocid is used in veterinary food up to 1 mg/Kg of body weight as an antiparasitic drug. This may represent up to 1.7 µM in tissues if biodistribution was even. The data presented here shows antitoxin activity between 1 and 10 µM. Mouse LD50 is 146 mg/Kg by oral route and 40 mg/Kg by the intraperitoneal route. Thus, there may be theoretically a therapeutic window for antitoxin use of lasalocid. 

The absence of organelle staining with lysotracker strongly suggests that lasalocid alters vesicular acidification. This could be explained by its broad ionophore activity that is capable of exchanging cations across membranes, including H^+^ [16]. Although the effect of lasalocid on organelles was studied on HeLa cells only, it is likely that similar effects would occur on other cell lines or types given the relative broad capacity of ionophores to interact with biomembranes.

At least two non-exclusive explanations could account for the antitoxin properties of lasalocid. The first one is that the toxins cannot find the acid conditions required for their conformational changes preceding translocation. For endolysosomal toxins (DT, CNF1, TcdB) membrane translocation occurs at the early and late endosome interface. For the retrograde trafficking toxins (Stx1, ETA), unfolding occurs prior to uptake by the endoplasmic-reticulum-associated protein degradation (ERAD) machinery and requires previous passage through the Golgi apparatus.

The second explanation for lasalocid’s antitoxin effect could be that the compartments in which toxins traffic or/and undergo translocation are altered in structure, function, or/and cell localization, as seen for endosomes and Golgi apparatus. These alterations could prevent toxin translocation. Several works from the ’70s and ’80s have shown by way of electronic microscopy that lasalocid leads to Golgi vacuolization in mammalian cells [15,30]. Here, we show that the Golgi apparatus is partially affected by lasalocid in HeLa cells, since GOLPH4 and GOLPH2 are both redistributed, whereas GM130 and giantin remain unchanged. GOLPH4 has been shown recently to interact with Stx1 and Stx2, and is essential to their retrograde trafficking [31,32]. GOLPH4 is known to cycle between endosomal compartments and the Golgi complex where it is prevalent in steady-state conditions. Alteration of pH with monensin, another ionophore altering vesicular trafficking and toxin action, prevents this cycling and promotes a relocalization of GOLPH4 to endosomes [33,34]. In parallel, one contribution links monensin to GOLPH2 endosomal redistribution, with a similar pattern seen here with lasalocid [34]. However, GM130 and giantin still exhibit a normal Golgi pattern after lasalocid exposure. Both of them are known to interact with, and participate to Golgi stacks organization and tethering [35,36]. Hence, lasalocid effects have consequences on Golgi/endosomes cycling proteins (GOLPH2, GOLPH4) but not on proteins involved in Golgi structures as stacked organelles.

Altogether, lasalocid has a broad-spectrum antitoxin activity and can be placed among trafficking inhibitors used as tools to study protein trafficking, organelle alterations and bacterial toxin effects.

## 4. Materials and Methods

### 4.1. Cell Cultures and Reagents

Human Umbilical Vein Endothelial Cells (PromoCell, Heidelberg, Germany) were grown on 0.2% (*w*/*v*) porcine skin gelatin-coated dishes at 37 °C under 5% CO_2_ in human endothelial SFM (11111-044, Life Technologies, Waltham, MA, USA), supplemented with 20% fetal bovine serum (12475-016, Life Technologies), 20 ng/mL bFGF (130-104, Miltenyi Biotec, Bergisch Gladbach, Germany), 10 ng/mL EGF (13247-051, Life Technologies), 1 μg/mL heparin (H3149, Sigma, St. Louis, MO, USA) and 100 U/mL penicillin—100 μg/mL streptomycin (15140-122, Life Technologies). All culture supports were pre-coated with 0.2% gelatin 15 min and then washed before cell seeding.

Human HeLa cells (ATCC, Manassas, VA, USA) and mouse L929 cells (ATCC) were maintained at 37 °C under 5% CO_2_ in Dulbecco’s modified Eagle’s medium (DMEM, 61965-026, Life Technologies), supplemented with 10% fetal bovine serum, 100 U/mL penicillin—100 µg/mL streptomycin, 0.1 mM non-essential amino acids (11140-035, Life Technologies). As indicated, some experiments were performed using DMEM without leucine (ME130029L1, Life Technologies).

The following products were purchased from the indicated commercial sources: [^14^C]-leucine and 96-well Cytostar-T^TM^ scintillating microplates were from Perkin-Elmer, Shelton, CT, USA; DMSO (D4540), donkey serum (D9663) and Resazurin (R7017) were purchased from Sigma; Dulbecco’s phosphate buffered saline (D-PBS, 14190-094) was from Life Technologies; Nunc 96-well optical bottom plates with coverglass base (164588), Corning 96-well clear bottom black plates (3603), epidermal growth factor-Alexa555 (EGF-A555, E35350), DQ Red bovine serum albumin (DQ-BSA, D12051), LysoTracker Green DND-26 (L7526), MitoTracker Green FM (M7514) and Hoechst 33342 dye (H33342, 14533) were from Thermo Scientific, Waltham, MA, USA. The following commercial antibodies were used in this study: anti-GOLPH2 (ab109628), anti-GOLPH4 (ab28049), anti-giantin (ab53542), anti-PDI (ab2792), anti-actin (ab3280) and Alexa 488-donkey anti-mouse (ab150109) from Abcam, Cambridge, UK; anti-EEA1 (C45B10) and anti-Rab9A (D52G8) from Cell Signaling, Danvers, MA, USA; anti-Mannose-6-phosphate receptor (M6PR, MA1-066) from Invitrogen, Waltham, MA, USA; anti-LC3B (LC3, L7543) from Sigma; Alexa 594-goat anti-mouse (115-585-146) from Jackson ImmunoResearch, Baltimore Pike West Grove, PA, USA. Paraformaldehyde (PFA, 15714) was from Electron Microscopy Sciences, Hatfield, PA, USA. Stx1 (#161) was purchased from List Biological Laboratories, Campbell, CA, USA and TcdB was produced and provided by M.R. Popoff (Institut Pasteur, Bactéries Anaérobies et Toxines unit, Paris, France). Exotoxin A was kindly provided by Bruno Beaumelle (UMR5236 CNRS, University of Montpellier, Paris, France).

### 4.2. Cytotoxicity Assay

A total of 2 × 10^4^ HeLa cells, L929 cells or HUVECs, were plated in 96-well clear bottom plate before lasalocid treatment overnight. Controls were either cells incubated with 10% DMSO (final dilution, positive control), or left untreated (negative control). Medium was then replaced by a dilution of resazurin in fresh medium (40 µg/mL corresponding to 160 nM, λ_Ex_ = 560 nm; λ_Em_ = 590 nm), for a 1 h incubation. Fluorescent signals were then measured with a Cytation 5 plate reader (BioTek), data were normalized, and curves were done using Prism v5 software (Graphpad Inc., San Diego, CA, USA). All values are expressed as means ± SEM, and represent duplicate experiments. The data shown are representative of two independent experiments.

### 4.3. Protein Biosynthesis Assay

A total of 2 × 10^4^ HeLa cells for Stx1 experiments, or 3 × 10^4^/well L929 for ETA experiments, were plated overnight in 96-well Cytostar-T scintillating microplates with scintillator incorporated into the polystyrene plastic. After 3 h of preincubation with various concentration of compounds (or 10% DMSO used as solvant) for indicated times at 37 °C, cells were challenged with increasing doses of Stx1 or ETA overnight in continued presence of compounds. Subsequently, the medium was removed and replaced with DMEM without leucine containing 10% fetal bovine serum, 2 mM L-glutamine, 0.1 mM non-essential amino acids, 100 U/mL penicillin—100 µg/mL streptomycin supplemented by 0.5 µCi/mL [^14^C]-leucine. Cells were grown for an additional 6 h at 37 °C under 5% CO_2_. Protein biosynthesis was then determined by measuring the incorporation of radiolabeled leucine into cells using a Wallac 1450 MicroBeta liquid scintillation counter (Perkin Elmer).

The mean percentage of protein biosynthesis was determined and normalized from duplicate wells. The data shown are representative of three independent experiments. All values are expressed as means ± SEM. Data were fitted using the nonlinear regression “dose-response EC_50_ shift equation” with Prism v5 software and the goodness of fit for toxin alone (carrier) or with drug was assessed by *r^2^* and confidence intervals.

### 4.4. TcdB Rounding Assay

HeLa cells were plated in 96-well optical bottom plate with coverglass base, treated overnight with lasalocid and 25 nM TcdB (added together), 25 nM TcdB alone, or left untreated. The next day, cells were fixed with 4% PFA for 15 min, and imaged with the Cytation 5 plate reader (BioTek, Winooski, VT, USA), objective 10×. The data shown are representative of three independent experiments. The percentage of rounded cells in each condition was calculated manually.

### 4.5. Live Cell Imaging

For EGF-A555 assays, HeLa cells were seeded in eight-wells non-separable LabTek, treated 5 h 30 min with 10 µM of lasalocid or left untreated, then EGF-A555 (λ_Ex_ = 555 nm; λ_Em_ = 565 nm), LysoTracker Green (λ_Ex_ = 485 nm; λ_Em_ = 520 nm) and H33342 were added for 30 min (final concentrations: 5 µg/mL, 100 nM, 1 µg/mL, respectively). Cells were washed with phosphate buffered saline (PBS) and immediately imaged with a Leica SP8X confocal microscope (Leica, Wetzlar, Germany), using a HC PL APO 63x/1.20 water CS objective.

Mitochondria staining protocol was identical, except that a solution containing MitoTracker Green FM (100 nM, (λ_Ex_ = 485 nm; λ_Em_ = 520 nm) and H33342 (1 µg/mL) was used instead of the EGF-A555/LysoTracker/H33342 mixture.

For DQ-BSA assays, HeLa cells were seeded in eight-wells non-separable LabTek, treated firstly with DQ-BSA (10 µg/mL) for 1 h. Supernatants were replaced by fresh medium containing 10 µM of lasalocid or not and this incubation lasted 6 h. Then, cells were washed with PBS and immediately imaged with a Leica SP8X confocal microscope, using a HC PL APO 63x/1.20 water CS objective. The data shown are representative of three independent experiments.

### 4.6. Immunostaining

HeLa cells were seeded in eight-wells LabTek, treated 6 h with lasalocid or left untreated, then fixed with 4% PFA 15 min. After PBS washes, permeabilization and saturation steps were performed for 1 h with a saturating buffer (1% donkey serum in D-PBS + 0.05% Tween 20) supplemented with 0.1% triton X-100. Cells were then labelled with indicated primary antibodies for 45 min, at following dilutions: anti-Rab9A: 200-fold dilution; anti-GOLPH2, GOLPH4, M6PR, EEA1, PDI: 500-fold dilution; anti-giantin: 1000-fold dilution. Washed cells were finally labeled with 500-fold diluted A488-anti-rabbit and A594-anti-mouse coupled secondary antibodies (λ_Ex_ = 490 nm; λ_Em_ = 520 nm and λ_Ex_ = 590 nm; λ_Em_ = 620 nm, respectively) for 45 min, before last washes and imaging with a Leica SP8X confocal microscope, using a HC PL APO 63×/1.40 oil CS2 objective. Nucleus staining with H33342 (1 µg/mL) was performed by mixing it with the secondary antibodies.

### 4.7. Imaging of HeLa Cells Stably-Expressing GFP-LC3B

We thank Dr. Aviva Tolkovsky (Cambridge Centre for Brain repair, Cambridge, UK) as well as Prof. Audrey Esclatine (University of Paris-Saclay, France) for providing HeLa cells stably-expressing GFP-LC3B [37]. Hela cells stably-expressing GFP-LC3B were maintained at 37 °C under 5% CO_2_ in RPMI medium (61870-010, Life Technologies), supplemented with 10% fetal bovine serum, 0.1 mM non-essential amino acids (11140-035, Life Technologies) and 500 µg/mL G418 (A1720, Sigma). 2.5 µM lasalocid or DMSO were added and incubated for 5 h. Live cells were directly observed for its green fluorescence with an inverted microscope (Ti-U, Nikon, Tokyo, Japan). The data shown are representative of two independent experiments.

### 4.8. Electrophoresis and Western Blot

Hela cells were seeded in 12-wells plate overnight, then treated 6 h with lasalocid at indicated concentrations or left untreated. Cells were lysed on ice, 15 min, with MPER lysis buffer complemented with protease inhibitor cocktail following the manufacturer recommendation. Lysate mixed with loading buffer containing β-mercaptoethanol were heated 5 min at 90 °C before SDS-PAGE, performed on 12% Mini-PROTEAN TGX precast gel (Bio-Rad, Hercules, CA, USA). Stain-free gel was imaged on a Gel Doc EZ imager (Bio-Rad), before transferring on a methanol-preactivated PVDF membrane.

Western blotting was done as followed: saturation step (PBS/0.05% Tween 20/1% milk) before staining of indicated proteins with antibodies diluted in saturating buffer (anti-LC3: 1/500, anti-actin: 1/2000), followed by HRP-coupled secondary antibody incubation (1/5000 in PBS/0.05% Tween 20) and then luminescence detection. The data shown are representative of two independent experiments.
